# The impact of duration and severity of obesity exposure on cardiometabolic health

**DOI:** 10.1007/s11695-024-07331-0

**Published:** 2024-06-04

**Authors:** Elisabeth S. van Ede, Simon W. Nienhuijs, Gijs H. Goossens, R. Arthur Bouwman, Marc P. Buise

**Affiliations:** 1 Department of Anesthesiology, Catharina Hospital, 5623 EJ Eindhoven, the Netherlands; 2 Department of Electrical Engineering, Signal Processing Systems, Eindhoven University of Technology, 5600 MB Eindhoven, The Netherlands; 3 Department of Surgery, Catharina Hospital, 5623 EJ Eindhoven, the Netherlands; 4 Department of Human Biology, NUTRIM School of Nutrition and Translational Research in Metabolism, Maastricht University Medical Center+, 6200 MD Maastricht, the Netherlands; 5 Department of Anesthesiology and Pain Medicine, Maastricht University Medical Center, 6229 HX Maastricht, the Netherlands

**Keywords:** Metabolic bariatric surgery, Obesity exposure, Obesity-related complications, Perioperative risk assessment

## Abstract

**Purpose:**

Duration and severity of exposure to excess adipose tissue are important risk factors for complications, but are generally not examined in conjunction. We developed a metric considering both factors to examine the relationship between obesity-related complications and parameters of cardiometabolic health in patients undergoing a metabolic bariatric procedure (MBS).

**Materials & Methods:**

Data from patients screened for primary MBS between 2017 and 2021 were analyzed. The Obesity Exposure score (OBES), based on self-reported years of life with a BMI ≥ 25 kg/m^2^, was calculated with increased weighting applied for higher BMI categories. Multivariate logistic regression analysis was performed, adjusting for multiple potential confounders.

**Results:**

In total, 2441 patients were included (76% female, age 42.1 ± 11.9 years, BMI 42.0 ± 4.9 kg/m^2^). OBES was positively related to myocardial infarction, atrial fibrillation and renal function loss (per 10 OBES-units: OR 1.31, 95%CI [1.11–1.52], p = 0.002; OR 1.23, 95% CI [1.06–1.44], p = 0.008; and OR 1.26, 95% CI [1.04–1.51], p = 0.02). OBES was negatively associated with obstructive sleep apnea syndrome (OSAS) (OR 0.90, 95% CI [0.83–0.98], p = 0.02). In patients without obesity-related complications, OBES was related to lower HbA1c and higher HDL-cholesterol levels (ß -0.5 95% CI [-0.08-.0.02] p < 0.001 and ß 0.02 [0.00–0.04] p = 0.01).

**Conclusion:**

OBES was related to myocardial infarction, atrial fibrillation and renal function loss in patients applying for MBS. OBES was negatively related to OSAS, possibly because undiagnosed years were not taken into account. In the absence of obesity-related complications, OBES was not related to metabolic blood markers. Our data may aid in improving perioperative risk assessments.

**Graphical Abstract:**

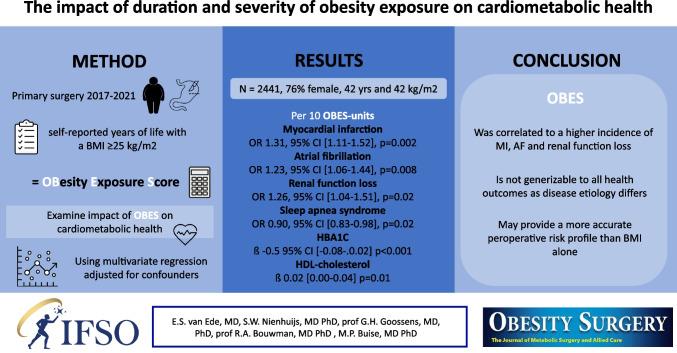

**Supplementary Information:**

The online version contains supplementary material available at 10.1007/s11695-024-07331-0.

## Background

The number of people living with obesity has risen globally in recent decades. More than 50% of adults are overweight and approximately 15% of adults in Europe (and in the Netherlands) is living with obesity [1]. Since obesity acts as a gateway to many noncommunicable diseases1, the incidence and burden of obesity-related complications have also increased [2, 3], as has the demand for metabolic-bariatric surgery. Assessing the health risks associated with obesity is an important part of treatment strategies and perioperative risk assessments. However, there is debate about the most appropriate method to determine how obesity contributes to this.

The risk of obesity-related complications may be influenced by several factors related to body fat, including age [4], the degree of obesity [5], weight gain [6, 7], the age of onset of obesity [8, 9], the duration of obesity [10–13], the obesity phenotype or fat mass distribution [12, 14, 15] and weight fluctuations [16]. Typically, these factors are examined independently of each other.

Estimating health outcomes using a composite measure has previously been applied using the so-called “fat-years” [13, 17] or “obese-years” [18]. Several studies have shown that combining the level and duration of obesity is better at predicting type 2 diabetes mellitus (T2DM) [18], all-cause mortality [11] and, to a lesser extent, cardiovascular disease (CVD) [19], than is BMI or duration of obesity alone. The patients in the cohort of these studies were 28–62 years old, had no history of disease or obesity, and were followed every two years for 48 years starting in 1948. In addition to the increase in the incidence and severity of obesity in recent decades, the percentage of older people and young people living with obesity has also increased, suggesting that patients are exposed to the detrimental consequences of excess adipose tissue for a longer period of time [17, 20]. As suggested earlier [18, 19], the use of a modern cohort as well as examining excess body weight higher than 25 kg/m2 instead of 30 kg/m2 would provide important complementary insights, as the incidence of chronic diseases increases with the degree of overweight and obesity [21]. Validating these results to patients living with obesity, research into the relationship between the composite measure of obesity duration and severity, which we refer to as the ‘OBesity Exposure Score’ (OBES), and obesity-related complications is needed in people who apply for a metabolic bariatric procedure. Moreover, such a cohort may be more homogeneous because the etiology of comorbidities may differ from that of patients without obesity.

Therefore, this study aimed to investigate the relationship between the duration and severity of exposure to obesity, quantified as OBES, and obesity-related complications and metabolic blood markers in a group of patients with an indication for a metabolic bariatric procedure in the Netherlands.

## Methods

### Data sources

This retrospective study used screening data from the Dutch Audit for Treatment of Obesity (DATO) registry combined with results from an OBES-questionnaire. Both were part of a screening trajectory for eligibility for a metabolic-bariatric procedure at an obesity center at a tertiary hospital in Eindhoven, the Netherlands, between January 2017 and January 2021.

The DATO registry is a nationwide quality registry for all metabolic-bariatric procedures [22]. Registry requires suitability for a metabolic bariatric procedure. The inclusion criteria were as follows: patients > 18 years of age with a body mass index (BMI) ≥ 40 or ≥ 35 kg/m^2^ and obesity-related complications: T2DM, hypertension, obstructive sleep apnea syndrome (OSAS), dyslipidemia, gastroesophageal reflux disease (GERD) and musculoskeletal pain.

The OBES-questionnaire is shown in Table 1. Patients had to compete the corresponding weight indicator at each life stage. The weight indices correspond with the weight range and BMI as defined by the World Health Organization [23]. The data were completed by using the Clinical Data Collector (CDC; CTcue B.V., Amsterdam, The Netherlands) [24]. This is a text mining-based tool for collecting structured and unstructured data from electronic patient files.

**Table 1:** OBES-questionnaire

Life stage (years)			Baby 0–1	Child 2–9	Teenager 10–17	Young adult 18–28	Adult 29–55	Older adult 56 +
Weight indicator	Weight range	Body Mass Index kg/m^2^						
Very fat (4)	Obesity class 2–3	> 35					x	x
Fat (3)	Obesity class 1	30—< 35				x		
Chubby (2)	Overweight	25—< 30			x			
Normal (1)	Healthy	18.5—< 25	x	x				
Skinny (0)	Underweight	< 18.5						

Example completed for a sample patient aged 60 years. Questionnaire translated from Dutch

### Patient Selection

The data of patients > 18 years old who had completed an OBES-questionnaire and who underwent a screening trajectory for a primary metabolic-bariatric procedure, including Roux-en-Y gastric bypass (RYGB), mini-RYGB, single anastomosis duodenal ileal bypass, sleeve gastrectomy, was extracted from the data sources.

The outcome measures included an overview of the patient's characteristics and the likelihood of an obesity-related complication per 10 OBES-units. For patients without obesity-related complications, the likelihood ratio of change in laboratory results per 10 OBES-units, was determined to be a cardiometabolic health indicator.

The collected patient characteristics included age, sex, BMI, waist circumference, and smoking and alcohol use status, as determined from the first screening appointment. *Obesity-related complications* were subdivided into cardiac (hypertension, myocardial infarction, diagnosis of heart failure, atrial fibrillation or atrial flutter (AF)) and noncardiac (T2DM, OSAS, renal function loss (GFR < 60 ml/min)) complications [25]. A complication was defined as being registered in the DATO registry or if a diagnosis or treatment for the complication had ever taken place in accordance with the EPF. To assess cardiometabolic health indicators, patients without obesity-related complications were selected from the cohort. The laboratory results included HbA1c, total cholesterol, high density lipoprotein-cholesterol (HDLc), the total cholesterol/HDLc ratio and creatinine levels, which were determined during the first screening appointment. It was assumed that these patients had not undergone any treatment that could influence their laboratory values. Patients with missing values were excluded from the analyses.

### OBesity Exposure Score

The OBES-score included the combination of the duration and severity of exposure to obesity and was calculated as follows. The sum of the number of years for which the patient indicated in the questionnaire that he or she was 'obese', i.e., a BMI > 30 kg/m^2^, was recorded. Additionally, weights were assigned to the following specific BMI categories: skinny (BMI < 18.5 kg/m^2^ = weight 0), normal (BMI 18.5- < 25 kg/m^2^ = weight 0), chubby (BMI 25–30 kg/m^2^ = weight 0.5), fat (BMI > 30 kg/m^2^ = weight 1.0) and very fat (BMI > 35 kg/m^2^ = weight 2.0). Assuming that the 10-year risk of health problems increases and, overall, for some conditions even doubles per increase in BMI category [2, 21].

$$OBES = years * 0.5 + years * 1.0 + years * 2.0$$

### Statistical analyses

Descriptive data are presented as the mean with standard deviation (SD) if normally distributed and as the median (interquartile range) if not normally distributed. Categorical data are presented as the absolute number and percentage. A multivariate regression model was used to analyze the association between *OBES* and obesity-related complications and cardiometabolic health status. Potentially confounding variables were included in the model if they were considered clinically relevant or if they tested statistically significant at p < 0.05 according to univariate logistic regression. The tested covariates included age, sex, *BMI, waist* circumference, smoking status, alcohol consumption*, diagnosis of* hypertension, myocardial infarction, heart failure, AF, T2DM, OSAS or renal function loss and HbA1c, total cholesterol, HDLc, total cholesterol/HDLc ratio and creatinine level [see Additional file 1]. The final multivariate regression model was obtained using stepwise backward elimination of nonsignificant covariates. For outcome variables less than 120 patients, a propensity weight was used to adjust for confounding variables [26, 27]. All variables in each model were tested for multicollinearity, effect modification or interaction with OBES*.*

## Results

In total, 2441 patients were included for in the analysis. A representation of patient characteristics in different age categories can be found in Table 2.

**Table 2:** Patient characteristics

Patient characteristics	Overall	Age 18–28 years	Age 29–55 years	Age ≥ 56 years
Patients	2441	414	1701	326
Age	42.1 ± 11.9	23.7 ± 3.0	43.2 ± 7.6	59.7 ± 3.1
Sex				
Male (%)	595 (24.4)	65 (15.7)	416 (24.4)	114 (35)
Female (%)	1846 (76)	349 (84.3)	1285 (75.5)	212 (65)
BMI (kg/m^2^)	42.0 ± 4.9	43.3 ± 4.4	41.8 ± 5.0	41.6 ± 4.9
Waist circumference (cm)	129.3 ± 13.3	127.6 ± 12.9	129.2 ± 13.6	132.2 ± 11.4
Smoker	461 (19)	126 (30.4)	296 (17.4)	39 (12)
Alcohol user	144 (6)	11 (2.7)	89 (5.2)	44 (13.5)
OBES	41.5 ± 16.8	24.4 ± 8.0	44.3 ± 15.3	48.7 ± 19.1
**No obesity-related complications**				
Patients	1238	331	846	61
Age	37.1 ± 11.0	23.7 ± 3.0	40.8 ± 7.4	59.3 ± 3.0
Sex				
Male	177 (14.3)	42 (12.7)	124 (14.7)	11 (18)
Female	1061 (85.7)	289 (87.3)	722 (85.3)	50 (82)
BMI (kg/m^2^)	42.4 ± 4.5	43.2 ± 4.2	42.2 ± 4.6	42.2 ± 4.3
Waist circumference (cm)	128.1 ± 13.0	126.9 ± 12.7	128.3 ± 13.3	131.8 ± 10.6
Smoker	249 (20.1)	92 (27.8)	150 (17.7)	7 (11.5)
Alcohol user	46 (3.7)	7 (2.1)	34 (4.0)	5 (8.2)
OBES	37.3 ± 15.9	24.4 ± 8.0	41.4 ± 15.1	50.0 ± 17.8

All included patients (upper part) and those who had no obesity-related complications (lower part). The data are presented as the mean ± standard deviation or number (%)

**Fig. 1:**
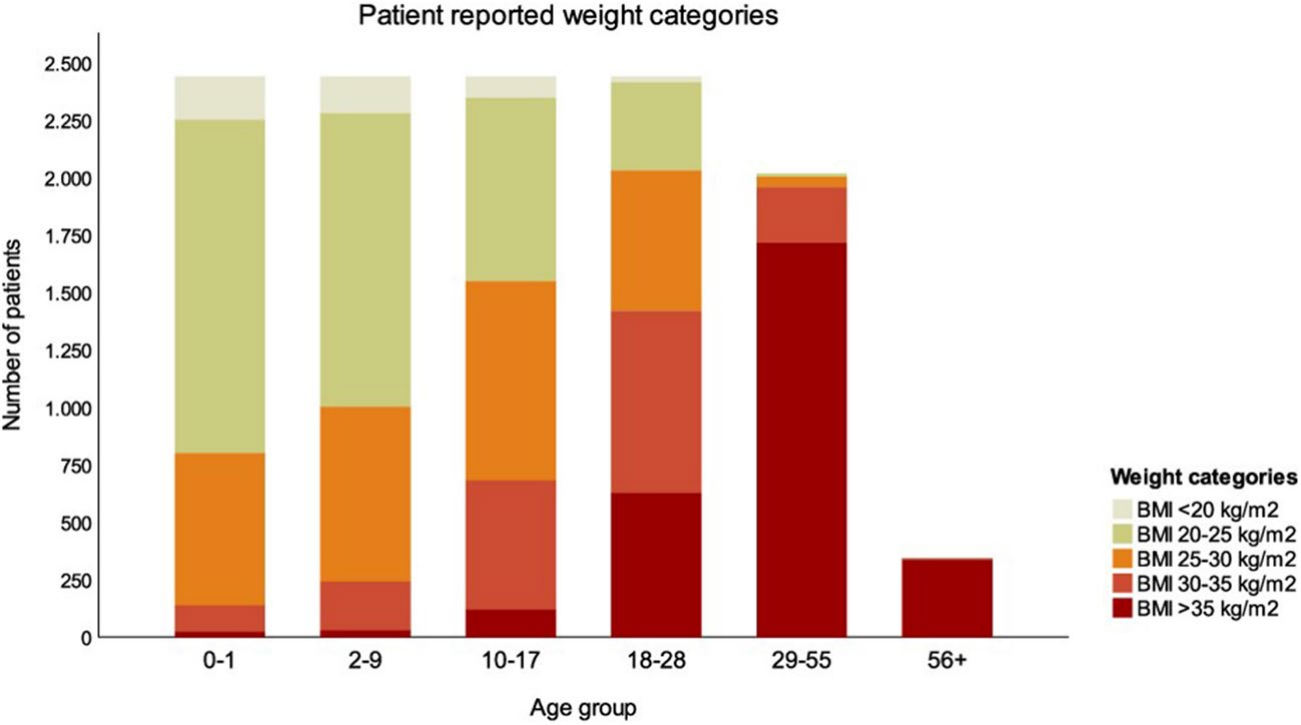
Patient-reported weight categories. Overview per age division of how often the patients assigned themselves a certain weight category

Figure 1 gives an overview per age division of how often the patients have assigned themselves a certain weight category. The number of individuals with a higher BMI increases with increasing age. More specifically, 83% (n = 2032) of the patients indicated that they had a BMI ≥ 25 kg/m^2^ and 58% a BMI ≥ 30 kg/m^2^ at the age of 18–28 years. For the 29–55 years age group, this was 98% and 97% respectively. In the oldest category (> 56 years), 100% of individuals reported a BMI ≥ 30 kg/m^2^.

The mean age at enrollment was greater for patients who had a BMI within the normal range during their baby years, childhood and adolescence (0–18 years) than those who had a BMI ≥ 25 kg/m^2^ (in 73% of the patients, n = 1777) or a BMI ≥ 30 kg/m^2^ (in 32% of the patients, n = 771) during those years. These values were 47.5 years (9.5 SD) and 40.0 years (12.1 SD) for individuals with a BMI ≥ 25 kg/m^2^ and 36.1 years (12.5 SD) for individuals with a BMI ≥ 30 kg/m^2^ respectively.

Table 3 shows the distribution of obesity-related complications in the total study population (upper) and the laboratory results for patients with no obesity-related complications (lower) across different age-categories and OBES (low/high based on mean values per age category). The obesity-related complication rate was overall greater in patients with a high OBES within the same age category, except for myocardial infarction in the youngest and AF, HF and OSAS in the oldest category. Additionally, the percentage of complications associated with a low OBES in any category tended to be greater than that associated with a high OBES in the younger category. Data of renal function loss were missing for 52 patients from the entire cohort. Laboratory values were missing for 35 patients without obesity-related complications.

**Table 3:** Distribution of obesity-related complications and laboratory results

Obesity-related complications	Age 18–28 years		Age 29–55 years		Age ≥ 56 years		Total
	OBES		OBES		OBES		
	Low < 24	High ≥ 24	Low < 44	High ≥ 44	Low < 49	High ≥ 49	
Atrial fibrillation	0	0	12 (1.4)	28 (3.2)	17 (9.3)	12 (8.5)	69 (2.8)
Myocardial infarction	2 (1.1)	0	27 (3.2)	42 (4.9)	19 (10.4)	15 (10.5)	105 (4.3)
Heart failure	0	0	4 (0.5)	16 (1.9)	10 (5.5)	6 (4.2)	36 (1.5)
Hypertension	11 (6.0)	12 (9.1)	205 (24.5)	349 (40.4)	109 (59.6)	90 (62.9)	785 (32.2)
Any cardial	12 (6.6)	12 (9.1)	222 (26.5)	370 (42.9)	119 (65)	99 (69.2)	843 (34.5)
OSAS	10 (5.5)	17 (7.3)	123 (14.7)	170 (19.7)	59 (32.2)	43 (30)	422 (17.3)
T2DM	10 (5.5)	13 (5.6)	100 (11.9)	174 (20.2)	60 (32.8)	47 (32.9)	403 (16.5)
Renal function loss	0	0	6 (0.7)	13 (1.5)	8 (4.4)	11 (7.7)	38 (1.6)
Any	28 (15.4)	43 (18.5)	332 (39.6)	505 (58.5)	144 (78.7)	116 (81.1)	1168 (47.8)
**Laboratory results**							
	OBES		OBES		OBES		
	Low < 24	High ≥ 24	Low < 47	High ≥ 47	Low < 49	High ≥ 49	
Total cholesterol	5.1 ± 1.1	4.8 ± 0.9	5.1 ± 1.0	5.1 [4.5–5.8]	5.1 ± 1.2	5.0 ± 1.2	5.0 ± 1.0
HDLc	1.0 [1.0–1.2]	1.1 ± 1.0	1.2 ± 0.3	1.2 ± 0.3	1.3 ± 0.4	1.2 ± 0.3	1.3 ± 0.3
Total cholesterol/HDLc ratio	4.9 ± 1.8	4.6 [3.5–5.4]	4.7 ± 1.5	4.3 [3.5–5.2]	4.1 [3.4–5.1]	4.1 [3.2–4.8]	4.3 ± 1.3
HbA1c	36 [32.8–43]	35 [32-40]	39 [35–46]	40 [37–50.3]	43 [38–51]	42 [38–54.5]	36.6 ± 5.6
Creatinin	68.1 ± 12.2	65.7 [12.6]	69 [60–79.5]	72 [62–83]	78.5 ± 15.2	75 [66–86]	67 ± 11.5

Upper: Number (%) of obesity-related complications in patients divided by age and OBES (low < mean (rounded) ≥ high). Lower: Mean ± SD or median (interquartile range [IQR]) laboratory results of patients with no obesity-related complications. Cholesterol, HDL-cholesterol (HDLc) and HbA1c are reported in mmol/ml, and creatinine is reported in µmol/l

Multivariate logistic regression adjusted for covariates [see Additional file 1] showed that OBES increased the likelihood of having suffered a myocardial infarction, being diagnosed with atrial fibrillation and showing signs of renal function loss. On the other hand, OBES decreased the likelihood of being diagnosed with OSAS (Table 4). For patients without obesity-related complications, OBES was significantly related to higher HDLc- and lower HbA1c levels (Table 5). Interaction analyses did not reveal differences between men and women.

**Table 4:** Results of multivariate logistic regression

*Obesity-related complications*	OR per 10 OBES-units (95% CI)	p value
Hypertension Myocardial infarction Atrial fibrillation Heart failure Any cardiac complication	0.980 (0.895–1.040) 1.305 (1.105–1.524) 1.231 (1.062–1.438) 1.207 (0.951–1.509) 0.970 (0.895–1.041)	0.380 0.002* 0.008* 0.120 0.400
T2DM OSAS Renal function loss Any complication	1.010 (0.932–1.094) 0.904 (0.834–0.980) 1.255 (1.041–1.509) 0.942 (0.869–1.020)	0.830 0.020* 0.020* 0.140

Relationship between OBesity Exposure Score (OBES) and obesity-related complications. CI = confidence interval. OSAS = obstructive sleep apnea syndrome

**Table 5:** Results of linear regression analysis

Laboratory value	Estimate (ß) per 10 OBES-units (95% CI)	p value
HbA1c Cholesterol total HDLc Creatinin	-0.50 (-0.8- -0.2) 0.01 (-0.04–0.1) 0.02 (0.00–0.04) -0.08 (-0.60–0.5)	< 0.001* 0.780 0.010* 0.770

Relationship between OBesity Exposure Score (OBES) and laboratory values levels of patients with no obesity-related complications. CI = confidence interval, HDLc = high-density lipoprotein cholesterol

## Discussion

The present study demonstrated that Obesity Exposure Score (OBES) was positively associated with different obesity-related complications such as MI, AF and renal function loss. This finding is in line with earlier research showing that a higher BMI was independently associated with a greater incidence of MI, regardless of metabolic health status [28], with chronic kidney disease [29] and new onset, progression, or recurrence of AF [30]. Abdullah et al. showed that a combination of the level and duration of exposure to obesity is a better estimate of CVD risk (in general) than BMI alone [19]. To our knowledge, the current study was the first to examine the relationship between each obesity-related comorbidity separately using the OBES, and hence extends the findings to previous populations in a different study.

A BMI measurement during a preoperative assessment, tends to be a static representation of a patient's body composition at that time. The amount and location of fat deposition have been shown to play a pivotal roles in multiple obesity-related complications [31–34]. For example, an elevated BMI and an increased neck circumference due to visceral adipose tissue accumulation around the neck and upper respiratory tract [35], are associated with a more frequent occurrence of OSAS [36]. The excess fat mass in people with obesity is strongly related to insulin resistance, impairments in lipid and glucose metabolism and low-grade systemic inflammation, thereby increasing the risk of obesity-related cardiometabolic complications, partly due to coagulation abnormalities, atherosclerosis, metabolic syndrome, insulin resistance, and T2DM [37]. Dysfunctional subcutaneous adipose tissue in obesity may also induce ectopic fat deposition [38, 39], including visceral adipose accumulation [40]. In addition, infiltration of adipose tissue in the epi- or pericardium is associated with conduction slowing, fibrosis and local inflammation contributing to the development of atrial fibrillation [41]. Since it often takes time for such conditions to manifest, long-term exposure to excess adipose tissue seems to play an important role in the development of obesity-related complications [12, 14, 42] and, hypothetically, in recovery.

Perioperative health and risk assessments are often performed using the American Society of Anesthesiologists Physical Status Score (ASA-PS score). Currently, patients with a BMI ≥ 40 kg/m^2^ receive a higher ASA-PS score than patients with a stable chronic disease such as hypertension or diabetes mellitus [43]. Cumulative exposure to obesity may play a role in the development of impaired metabolic health [12] and cardiovascular comorbidities [19]. The presence or severity of comorbidities may be enhanced but can also be masked by a nonactive lifestyle or low exercise tolerance [13]. Therefore, the addition of OBES in addition to BMI alone may be considered for perioperative health assessments.

In the present study, we did not find a positive association between OBES and OSAS incidence. OSAS is apparently less influenced by the cumulative duration of obesity than by factors such as BMI, height or neck circumference [44], despite the link between obesity and visceral fat accumulation. Because the etiology and therefore the way in which obesity contributes may differ between chronic diseases, OBES may not be generalizable to all obesity-related comorbidities. Nevertheless, it should be kept in mind that OSAS is often underdiagnosed [45], which may have influenced the results.

Contrary to our expectations, the present data did not show that OBES was related to the occurrence of hypertension, T2DM or elevated HbA1c. Previous studies have shown that earlier onset and cumulative exposure to obesity (degree and/or duration) result in a greater incidence of glucose intolerance, a higher HbA1c level or T2DM [10, 18, 46, 47]. In particular, longer periods of obesity can cause a β-cell dysfunction in the pancreas, resulting in insufficient insulin secretion to maintain normal glucose homeostasis. There are a number of explanations for these discrepant findings. The patients in our study may have been treated for hyperglycemia, leading to surgery. We have not been able to retrieve this information from the registries. Furthermore, older patients with a lower OBES were more often diagnosed with a complication than patients with a higher OBES from a younger age category. This finding suggested that in addition to exposure to obesity, older age may also play an important role in the development of complications.

A strength of the present study is that it is the first to separately examine the relationship of each obesity-related comorbidity using OBES. Second, the risk of obesity-related complications was calculated in a bariatric population. When this risk was calculated with respect to patients without obesity, as has been done in other studies, a reduction in risk requires weight loss to a BMI normalization. In addition to the fact that weight loss after bariatric surgery is defined as a % loss of excess or total weight, the vast majority of patients often do not achieve weight 'normalization' in the longer term [48, 49]. Finally, we were able to use data from a large cohort with a complete dataset on the progression of estimated weight from all life stages, including early childhood and adolescence up to metabolic-bariatric surgery. This study has several limitations. OBES was based on self-reported data regarding BMI. Patients frequently indicated that they did not consider themselves to be living with obesity in the years before the surgical indication, which may have resulted in an underestimation of the OBES scores. Nevertheless, it is questionable whether objective data can be obtained in clinical practice. Another limitation was the lack of information on the time to diagnosis. Childhood and adolescent obesity was indicated more often by younger patients than by older patients. However, OBES did not take any contributing factors to the timing of the onset of obesity into account, which could have influenced the results.

## Conclusions

In patients applying for a metabolic bariatric procedure, OBES was related to myocardial infarction, atrial fibrillation and renal function loss in a gradual manner. This could be relevant for anesthesia risk assessments. A negative relationship was found for OSAS incidence, possibly because undiagnosed years of OSAS were not considered. In addition, in the absence of obesity-related complications, OBES was not related to metabolically unhealthy blood markers. Due to differences in disease etiology, OBES may not be generalizable to all obesity-related complications. Future research can be conducted to evaluate the influence of OBES on the success rate of weight loss and resolution of obesity-related complications after bariatric surgery. To further investigate the usefulness of adding a score in preoperative assessments, it can first be investigated whether there is a difference between the incidence of perioperative complications in bariatric patients in the same ASA class based on weight alone or on the basis of (metabolic) comorbidities.

### Supplementary Information

Below is the link to the electronic supplementary material.

Supplementary file1 (XLSX 12 KB)

## Data Availability

Data are available from the corresponding author upon request.
